# Syncope in Pulmonary Thromboembolism: A Cross-Sectional Analysis of Risk Factors and the Prognostic Value of Syncope

**DOI:** 10.3390/jcm14072501

**Published:** 2025-04-06

**Authors:** Songul Ozyurt, Neslihan Ozcelik, Elvan Senturk Topaloglu, Abdurrahman Kotan, Aziz Gumus, Unal Sahin

**Affiliations:** 1 Department of Chest Diseases, Faculty of Medicine, Recep Tayyip Erdogan University, Rize 53100, Turkey; songul.ozyurt@erdogan.edu.tr (S.O.); neslihan.ozcelik@erdogan.edu.tr (N.O.); aziz.gumus@erdogan.edu.tr (A.G.); unal.sahin@erdogan.edu.tr (U.S.); 2 Department of Chest Diseases, Erzurum Regional Training and Research Hospital, Erzurum 25100, Turkey; kotanmd128@gmail.com

**Keywords:** pulmonary thromboembolism, syncope, mortality

## Abstract

**Background/Objectives**: This study investigated the frequency of syncope, factors associated with syncope, and the relationship between syncope and mortality in patients with pulmonary thromboembolism (PTE). **Methods**: This study was planned as single-center retrospective and cross-sectional research. The PTE diagnosis was confirmed by partial or complete filling defects in at least one branch of the pulmonary circulation seen on pulmonary spiral computed tomography angiography. Patients’ demographic data, symptoms, location of pulmonary embolism, Simplified Pulmonary Embolism Severity Index (sPESI) risk group, European Society of Cardiology (ESC) risk group, in-hospital mortality rate, and 30-day mortality rate were recorded. The presence of syncope and associated factors as well as the relationship between syncope and mortality were investigated. **Results**: This study included 589 consecutive patients diagnosed with PTE. The mean age was 70 ± 15 years, and 58.7% of the patients were women. Syncope was detected in 12.4% of the patients. Female sex, pulse rate, thrombosis in the main pulmonary artery, and right ventricular dysfunction on an echocardiogram were more prevalent in the syncope group. In-hospital mortality was 2.1 times higher in the syncope group. Elevated troponin levels increased the occurrence of syncope by 4.9-fold, whereas the presence of thrombosis in the main pulmonary artery and signs of right ventricular failure increased syncope occurrence by 4.3- and 3.1-fold, respectively. **Conclusions**: In the presence of syncope, patients with pulmonary thromboembolism, embolism in the main pulmonary arteries, high troponin values, right heart failure, and a high sPESI risk group should be carefully assessed and closely monitored for mortality, and reperfusion therapy should be considered as necessary.

## 1. Introduction

Pulmonary thromboembolism (PTE) can have a range of clinical manifestations from asymptomatic status to shock or sudden death. Shortness of breath and chest pain are considered the most prevalent symptoms [1]; however, many patients, including those who are critically ill, may present with mild or non-specific symptoms. Although rarely seen in PTE, presyncope, syncope, and hemodynamic collapse can lead to mortality. Since syncope is often caused by transient hypotension, it may be an indicator of high-risk pulmonary embolism in clinical practice. In addition to a meta-analysis that reported the prevalence of PTE as <1% in patients who presented with syncope [2], a higher prevalence rates of syncope have been reported [3].

Syncope has been defined as a transient loss of consciousness with rapid onset, short duration, and spontaneous recovery in cases where obvious causes like epileptic seizure, stroke, and head trauma are excluded [4]. The prevalence of embolism in patients who presented to hospital with a syncope attack was 17% according to a study [3]. Moreover, the same study reported the frequency of syncope in patients with pulmonary embolism as 24.7%. A recent study reported that syncope was a symptom that occurred in 10–20% of patients with PTE [5]. Although some patients with PTE who present with syncope might be normotensive, a study reported that the mortality risk could reach up to 30% if hemodynamic instability accompanied this symptom [5]. Therefore, syncope is considered a rare but important symptom in PTE.

Possible mechanisms of syncope due to pulmonary embolism include right ventricular failure and left ventricular filling disorder associated with an occlusion in >50% of the pulmonary arteries, resulting in a sudden decrease in cardiac output and cerebral blood flow, arrhythmias caused by overstraining of the right ventricle, and vasovagal reflex caused by embolism [6].

Previous studies investigated the prevalence of embolism in patients who presented with syncope and the frequency of syncope in patients with pulmonary embolism. However, their reports on the effect of syncope on the prognosis remain contradictory and debated. This study investigated the frequency of syncope, risk factors associated with syncope, and the prognostic value of syncope in relation to mortality in patients with PTE.

## 2. Materials and Methods

### 2.1. Study Design and Study Population

This retrospective, single-center, cross-sectional study included 589 consecutive patients with a confirmed diagnosis of pulmonary thromboembolism between 1 January 2018 and 1 January 2023, a 5-year period. The PTE diagnosis was confirmed by partial or complete filling defects in at least one branch of the pulmonary circulation seen on pulmonary spiral computed tomography angiography (CTA) (Toshiba Alexion TSX-034A, Shimoishigomi, Otawara-Shi, Toschigi-Ken, Japan). Demographic data, comorbidities, symptoms at presentation, predisposing factors, Wells’ clinical score, location of embolus on CTA, laboratory results, lower extremity Doppler ultrasonography (LOGIQ E9 US system GE Heathcare, Milwaukee, WI, USA) results, echocardiography (Philips iE33 Medical ultrasound Systems device, Andover, MA, USA) results, initial and maintenance treatment, in-hospital mortality status, and mortality status 1 month upon discharge were recorded. sPESI and risk group classification were performed according to the ESC 2019 acute pulmonary embolism guidelines [1].

sPESI was calculated by assigning one point for the occurrence of each of the following variables at presentation: age >80 years, a history of cancer, a history of chronic cardiopulmonary disease, heart rate ≥110 beats per minute (bpm), systolic blood pressure ˂100 mm Hg, and arterial oxyhemoglobin saturation ˂90% measured at the time of PE diagnosis. Patients were classified as at low risk (0 points) or at high risk (≥1 point) pursuant to sPESI [1].

The occurrence of right ventricular dysfunction (RVD) was confirmed by an echocardiogram procedure. A right ventricular (RV)/left ventricular ratio of >0.9 was defined as RV hypokinesia based on the criteria defined by the American Heart Association [7].

Syncope was defined as a sudden, transient loss of consciousness due to transient cerebral hypoperfusion. It typically occurred following a postural change, such as standing up from a seated or lying position, and resolved spontaneously [3,8]. Syncope characteristics were obtained either from the patients themselves or from eyewitnesses. All patients underwent a comprehensive assessment to exclude potential causes of syncope unrelated to pulmonary embolism.

The present study investigated the prevalence of syncope, factors affecting the presence of syncope, and the effect of syncope on mortality in patients with PTE. The primary and secondary endpoints of the study were in-hospital mortality and 30-day mortality, respectively. This study was approved by the local institutional ethics committee (2024/118).

### 2.2. Inclusion Criteria

Patients aged >18 years with a confirmed diagnosis of pulmonary thromboembolism via CTA.

Patients with complete clinical and demographic data

Patients with complete data; Patients who were followed-up 30 days after discharge and confirmed alive.

### 2.3. Exclusion Criteria

Patients who had suspected pulmonary thromboembolism but could not undergo computed tomography angiography (CTA) for confirmation were excluded from this study. Additionally, to eliminate potential confounders, patients with cardiac conditions known to cause syncope were not included. These conditions included bradycardia, supraventricular and ventricular tachycardia, aortic stenosis, acute myocardial infarction or ischemia, hypertrophic cardiomyopathy, cardiac tumors such as atrial myxoma, pericardial diseases including tamponade, congenital coronary artery anomalies, and prosthetic valve dysfunction. Furthermore, patients with a history of head trauma or intracranial hemorrhage, as well as those who experienced syncope due to hypotension induced by antihypertensive medication use, were excluded.

Patients with missing data; Patients who were lost to follow-up 30 days after discharge.

### 2.4. Data Analysis

The Statistical Package for the Social Sciences (IBM SPSS; SPSS Inc., Chicago, IL, USA) software version 21 was used for statistical analyses. The hypothesis of normal distribution of continuous variables was tested by the Kolmogorov–Smirnov test. Continuous and categorical variables were presented as a median (interquartile range) and number (%), respectively. The Mann–Whitney U test was used to compare the two groups. The Chi-Squared test was used to compare the categorical variables. Univariate and multivariate regression analyses were performed to determine the factors associated with syncope. A *p* value of <0.05 was considered statistically significant.

## 3. Results

**Figure 1:**
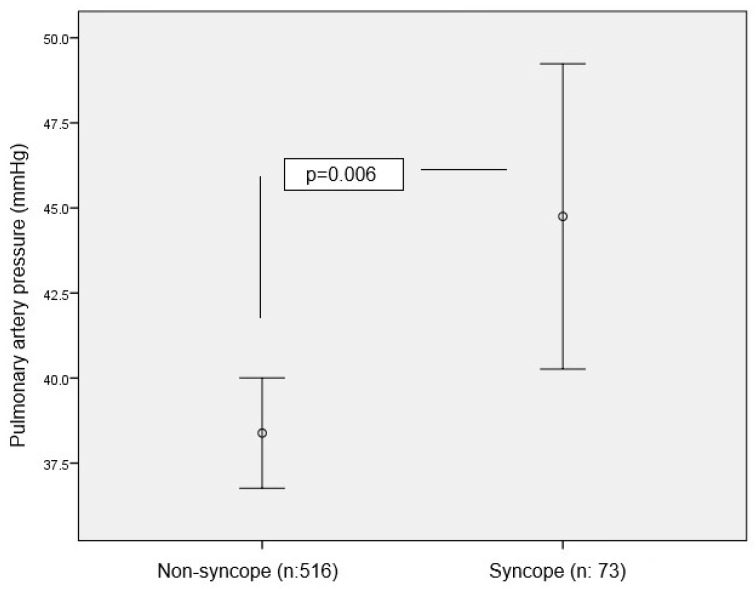
Error bar plot of pulmonary artery pressure between syncope and non-syncope groups.

A total of 589 consecutive patients with PTE (mean age = 70 ± 15 years; range = 18–99), including 346 (58.7%) women, were included in this study. PTE was diagnosed upon thoracic CT angiography in all patients. The patients were evaluated vis-a-vis syncope status at presentation. The numbers of patients with and without syncope were 73 (12.4%) and 516 (87.6%), respectively. Upon a comparison of patients with and without syncope, the majority of the patients were women, the pulse rate was higher, thrombosis in the main pulmonary artery was significantly more prevalent, and RVD was more common on ECHO in patients with syncope. Pulmonary arterial pressure was significantly higher in the group of patients with syncope (Figure 1). Moreover, in-hospital mortality was significantly higher in patients with syncope. A comparison of patients with and without syncope based on demographic, clinical, laboratory, and imaging methods is given in Table 1.

Univariate logistic regression analyses were performed to determine the factors associated with syncope (Table 2). Female sex, increased pulse rate, increased d-dimer, thrombosis in the main pulmonary artery, increased pulmonary artery pressure, RVD on ECHO, troponin elevation, and high-risk status on sPESI were associated with the development of syncope. The most prominent factors that affected the development of syncope were troponin elevation (OR: 4.988, *p* < 0.001), thrombosis in the main pulmonary artery (OR: 4.357, *p* < 0.001), and RVD seen on ECHO (OR: 3.185, *p* < 0.001). Furthermore, an occurrence of syncope was associated with in-hospital mortality. In-hospital mortality was significantly higher in patients with syncope than in those without (OR: 2.170, *p*: 0.035)

A multivariate logistic regression analysis was performed to determine the independent factors that affected the development of syncope (Table 3). Pulse rate increase (OR: 1.029, *p* = 0.005), thrombosis in the main pulmonary artery (OR: 5.609, *p* < 0.001), and RVD on ECHO (OR: 2.704, *p* = 0.044) were independent variables for the development of syncope. The thrombosis in the main pulmonary artery was the most effective factor.

## 4. Discussion

Pulmonary embolism is a condition that can present with a variety of symptoms and clinical signs, most commonly dyspnea and chest pain. However, it is also a potential and significant cause of syncope. Syncope is the initial presentation in approximately 10% of patients with pulmonary embolism. The rate of syncope was 12.4% in the present study. The prevalence of syncope was significantly higher in patients with thrombus in the main pulmonary artery and troponin elevation as well as in patients who were classified as at high risk based on sPESI. In-hospital early mortality was higher in the patients with syncope.

Syncope was first reported in patients with PTE in the 1970s and was associated with an increased clot burden, RV strain on an electrocardiogram, and hypotension [9]. The syncope rate was reported as 10.4% in a study by Zhang S. et al. [10] and as 5.5% in another multicenter study [11]. A study by Richmond C et al. [12] and Lee YH et al. [13] reported the frequency of syncope as 9.8% and 4.2%, respectively. Moreover, the syncope rate was reported as 16% in another study of 588 patients [14]. A meta-analysis of 29 studies on a large number of patients reported the prevalence of syncope as 16.9% [15]. However, in the present study, the syncope rate was 12.4%.

A study by Dzudovic B. et al. reported that the prevalence of syncope was higher in men [14]. Another study by Zhang S. et al. [10] reported that the prevalence of syncope was higher in the women, similar to the present study. The sex-based differences in the autonomic nervous system in women might have caused to the higher prevalence of syncope in this sex [16]. Moreover, the left ventricle is smaller and stiffer in women. Therefore, a relatively greater reduction in stroke volume might occur, making the women more susceptible to syncope [17].

Tachycardia, hypotension, and impaired cerebral perfusion may occur as a result of a left ventricle affected by massive PE acute RV failure. We believed that hypotension occurred by means of the Bezold–Jarisch reflex mechanism because the thromboembolism obstructed the main pulmonary artery [18]. Therefore, cardiac damage caused by a severe clot burden, decreased cardiac output due to right heart dilatation, and transient hypoperfusion of the brain were responsible for the development of syncope. In the present study, the occurrence of a thrombus in the main pulmonary artery, troponin elevation, and RV failure on ECHO were associated with a 5.6, 2.5, and 2.7-fold increases in the prevalence of syncope, respectively.

An elevated troponin level reflects a higher RV afterload because of the central pulmonary artery occlusion. Thus, RV failure is considered a contributory factor for the development of syncope. Previous studies also reported that syncope in patients with acute PTE was associated with an increased prevalence of central embolism, RVD, and troponin positivity [19,20].

There are contradictory data regarding the prognostic significance of syncope in acute pulmonary embolism. Therefore, it has been generally considered a poor prognostic factor. In recent years, several studies have reported that syncope had no effect on early mortality [7,10,19]. However, another study reported that syncope was associated with mortality in high-risk patients with pulmonary embolism [6]. In the present study, the in-hospital mortality rate was higher in patients with syncope across the whole cohort group and syncope increased the in-hospital mortality rate by approximately 2.2-fold.

As for the 30-day mortality rate after discharge, Dzudovic B. et al. reported that syncope was associated with 30-day mortality only in women [14]. Iqbal U et al. reported that the 30-day mortality rate was higher in patients with syncope regardless of sex [7]. In another study, there was no association between the 30-day mortality rate and the occurrence of syncope [21]. In the present study, there was no association between the occurrence of syncope and the 30-day mortality rate.

However, careful monitoring and closer follow-up of patients with syncope at baseline and the application of more aggressive therapeutic approaches might have accounted for the contradictory results regarding mortality in patients with syncope. Furthermore, it was suggested that syncope might not be induced by the same mechanism in every patient, which might cause different outcomes [10].

Our study highlights the importance of considering pulmonary embolism (PE) as a potential cause of syncope. Current risk stratification scores for syncope, such as the Osservatorio Epidemiologico sulla Sincope nel Lazio (OESIL) and the Canadian Syncope Risk Score (CSRS), have been widely used to predict short-term adverse cardiac events in syncope patients. However, recent studies suggest that while these scores demonstrate high sensitivity, they often lack specificity in identifying patients who require inpatient cardiovascular evaluation [22]. This limitation underscores the need for improved risk assessment tools that can better distinguish high-risk patients. Improved risk stratification models may aid in more accurate clinical decision-making and reduce missed PE diagnoses in syncope patients.

The significance of syncope in pulmonary embolism has not been fully elucidated. While the occurrence of syncope is more commonly expected in massive cases, a study reported that even in hemodynamically stable patients, those with syncope accompanied by right ventricular dysfunction experienced higher rates of in-hospital adverse events. It was highlighted that these patients were at risk of complications such as the need for mechanical ventilation, hemodynamic instability, inotropic support, reperfusion therapy, and in-hospital mortality [23]. In the study by Jichun Liu et al., it was suggested that although patients were initially diagnosed with intermediate-risk pulmonary embolism, the development of syncope during follow-up could indicate in-hospital adverse events [24]. Therefore, we emphasize the need for careful monitoring of patients presenting with syncope at admission or developing syncope during follow-up, as syncope remains a significant concern in pulmonary embolism.

### Strengths and Limitations

The limitation of the present study is its single-center and retrospective nature. However, among the patients presenting with syncope, the likely causes of syncope other than embolism and especially neurological diseases, were excluded via central imaging, and the diagnosis of pulmonary thromboembolism was confirmed via pulmonary CT angiography in all patients. Those are considered as this study’s strengths.

## 5. Conclusions

Pulmonary embolism should be considered in the differential diagnosis if syncope cannot be explained by another cause. Although syncope is not considered one of the prevalent symptoms of pulmonary thromboembolism, it should be noted that it may be associated with embolism in the main pulmonary arteries, higher troponin values, right heart failure, a higher sPESI risk group classification, and early mortality. Therefore, patients with syncope should be evaluated for the occurrence of these adverse conditions and such patients should be closely monitored for reperfusion therapy.

## Figures and Tables

**Table 1:** A comparison of patients with pulmonary thromboembolism with and without syncope based on demographic, clinical, laboratory, and imaging methods.

	Patients with Syncope (*n* = 73)	Patients Without Syncope (*n* = 516)	*p* Value
Age (Year)	73 (62–83)	77 (65–87)	0.694
Sex (F/M)	54/19	292/224	0.005
Chest pain (%)	27 (19.7)	165 (31.9)	0.393
Smoking (smokers and ex-smokers) (%)	12 (16.4)	108 (20.9)	0.372
Cancer (%)	7 (9.6)	104 (20.2)	0.031
Dyspnea (%)	55 (75.3)	448 (88.8)	0.009
Hemoptysis (%)	2 (2.7)	27 (5.2)	0.357
Change in consciousness (%)	25 (34.2)	68 (13.2)	<0.001
Systolic BP	110 (90–120)	120 (110–124)	0.031
Diastolic BP	70 (60–80)	70 (60–80)	0.578
Leg swelling (%)	19 (26.0)	104 (20.2)	0.248
Pulse	116 (100–130)	98 (86–108)	0.002
Respiratory rate	22 (19–25)	25 (19.5–26)	0.852
Temperature (°C)	36.7 (36.6–36.9)	36.7 (36.4–36.9)	0.453
D-Dimer	3.92 (1.92–6.57)	3.18(1.72–4.95)	0.030
CRP	3.30 (1.55–5.45)	5.04 (1.70–9.76)	1.161
Creatinine	0.90 (0.81–1.18)	0.81 (0.70–1.10)	0.016
pH	7.42 (7.39–7.46)	7.44 (7.40–7.47)	0.335
PCO_2_	32.5 (27–35.5)	33 (28–36.1)	0.284
PO_2_	57 (50.7–71.1)	65 (57–78)	0.135
SaO_2_	92 (86.4–94.6)	93.4 (89–95.6)	0.051
Thrombosis in the main pulmonary artery (%)	52 (71.2)	187 (36.2)	<0.001
Troponin elevation (%)	60 (82.2)	248 (48.1)	<0.001
RVD on ECHO (%)	45 (61.6)	168 (32.6)	<0.001
DVT (%)	34 (46.6)	209 (40.5)	0.612
sPESI (high risk) (%)	64 (87.7)	361 (69.9)	0.002
Pulmonary artery pressure	45 (33–58)	35 (24–49)	0.006
Thrombolytic (%)	23 (31.5)	29 (5.6)	<0.001
In-hospital mortality (%)	11 (15.1)	39 (7.6)	0.031
Length of hospital stay (days)	7 (5)	7.5 (5)	0.261
Recurrent VTE (%)	7 (9.6)	36 (6.9)	0.422

Data are presented as number, percentage, and median (interquartile range). CRP: C-reactive protein, PCO_2_: partial pressure of carbon dioxide, PO_2_: partial pressure of oxygen, SaO_2_: oxygen saturation, ECHO: echocardiography, RVD: right ventricular dysfunction, DVT: deep vein thrombosis, sPESI: Simplified Pulmonary Embolism Severity Index, VTE: venous thromboembolism.

**Table 2:** A univariate logistic regression analysis of factors associated with syncope.

Variables	OR	95% Confidence Interval	*p* Value
Sex (Female)	2.180	1.257–3.782	0.006
Dyspnea	0.464	0.257–0.837	0.011
Cancer	0.420	0.187–0.943	0.035
Change in consciousness	3.431	1.987–5.927	<0.001
Systolic BP	0.990	0.978–1.003	0.124
Pulse	1.023	1.010–1.036	0.001
D-Dimer	1.137	1.037–1.246	0.006
Creatinine	1.801	0.949–3.417	0.072
Thrombosis in the main pulmonary artery	4.357	2.545–7.458	<0.001
Troponin elevation	4.988	2.672–9.309	<0.001
RVD on ECHO	3.185	1.908–5.315	<0.001
sPESI (high risk)	3.053	1.482–6.290	0.002
Pulmonary artery pressure	1.025	1.007–1.043	0.007
In-hospital mortality	2.170	1.057–4.456	0.035

BP: blood pressure, sPESI: Simplified Pulmonary Embolism Severity Index, ECHO: echocardiography, RVD: right ventricular dysfunction.

**Table 3:** A multivariate logistic regression analysis of factors associated with syncope.

Variables	OR	95% Confidence Interval	*p* Value
Sex (female)	0.775	0.352–1.708	0.528
Cancer	0.349	0.093–1.306	0.118
Change in consciousness	2.228	0.962–5.157	0.061
Pulse	1.029	1.009–1.050	0.005
D-Dimer	1.056	0.925–1.206	0.422
Thrombosis in the main pulmonary artery	5.609	2.364–13.307	<0.001
Troponin elevation	2.491	0.987–6.282	0.053
RVD on ECHO	2.704	1.029–7.104	0.044
Pulmonary artery pressure	0.907	0.941–1.000	0.053

ECHO: echocardiography, RVD: right ventricular dysfunction.

## Data Availability

All data generated or analyzed during this study are included in this article. The data will be available upon reasonable request (contact persons: drsongul@gmail.com and elvansenturk@ktu.edu.tr).
